# Roles for Stress Response and Cell Wall Biosynthesis Pathways in Caspofungin Tolerance in *Cryptococcus neoformans*

**DOI:** 10.1534/genetics.119.302290

**Published:** 2019-07-02

**Authors:** Kaila M. Pianalto, R. Blake Billmyre, Calla L. Telzrow, J. Andrew Alspaugh

**Affiliations:** * Department of Medicine, Duke University School of Medicine, Durham, North Carolina 27710; † Department of Molecular Genetics and Microbiology, Duke University School of Medicine, Durham, North Carolina 27710

**Keywords:** antifungal, chitin, calcineurin, signalosome, Ras

## Abstract

Limited antifungal diversity and availability are growing problems for the treatment of fungal infections in the face of increasing drug resistance. The echinocandins, one of the newest classes of antifungal drugs, inhibit production of a crucial cell wall component. However, these compounds do not effectively inhibit the growth of the opportunistic fungal pathogen *Cryptococcus neoformans*, despite potent inhibition of the target enzyme *in vitro*. Therefore, we performed a forward genetic screen to identify cellular processes that mediate the relative tolerance of this organism to the echinocandin drug caspofungin. Through these studies, we identified 14 genetic mutants that enhance caspofungin antifungal activity. Rather than directly affecting caspofungin antifungal activity, these mutations seem to prevent the activation of various stress-induced compensatory cellular processes. For example, the *pfa4*Δ mutant has defects in the palmitoylation and localization of many of its target proteins, including the Ras1 GTPase and the Chs3 chitin synthase, which are both required for caspofungin tolerance. Similarly, we have confirmed the link between caspofungin treatment and calcineurin signaling in this organism, but we suggest a deeper mechanism in which caspofungin tolerance is mediated by multiple pathways downstream of calcineurin function. In summary, we describe here several pathways in *C. neoformans* that contribute to the complex caspofungin tolerance phenotype in this organism.

INVASIVE fungal diseases primarily affect people with immune system defects, resultingin significant morbidity and mortality in these vulnerable patient populations (Park *et al.* 2009; Pyrgos *et al.* 2013; Rajasingham *et al.* 2017). Limited therapeutic options and availability are major challenges for effective treatment of systemic fungal infections, particularly in regions where fungal infection rates are highest (Loyse *et al.* 2013; Perfect and Bicanic 2014). Historically, it has been difficult to identify novel antifungal agents that are not also toxic to humans, since many cellular processes are highly conserved between humans and fungi. In the search for novel antifungal drugs, identification of fungi-specific cellular processes has been a major focus. The fungal cell wall represents a key structure for fungal viability, growth, and host evasion (Latgé 2007; Doering 2009; Gow and Hube 2012; O’Meara *et al.* 2013; Esher *et al.* 2018). Thus, compounds that target the production and maintenance of the fungal cell wall, with little to no effect on the human host, would make exciting and specific antifungal agents.

Echinocandins are cell wall-targeting antifungal compounds that have been identified and synthesized from natural products (Denning 2003; Letscher-Bru and Herbrecht 2003). These compounds inhibit the synthesis of β-1,3-glucan, a crucial cell wall component for many fungi (Taft *et al.* 1988; Kurtz *et al.* 1994; Kurtz and Douglas 1997; Maligie and Selitrennikoff 2005). Echinocandin antifungals, such as caspofungin, micafungin, and anidulafungin, are used extensively in clinical settings for the treatment of infections caused by diverse fungi. However, echinocandins do not have potent antifungal activity against the fungal pathogen *Cryptococcus neoformans*, whose growth is only inhibited at high levels of caspofungin that are not clinically achievable in patients (Abruzzo *et al.* 1997; Bartizal *et al.* 1997; Espinel-Ingroff 1998). The fact that this drug is so ineffective against this fungus is surprising for a number of reasons. First, the gene that encodes the β-1,3-glucan synthase catalytic subunit in *C. neoformans*, *FKS1*, is essential in this organism (Thompson *et al.* 1999). Additionally, the *C. neoformans* enzyme is highly sensitive to caspofungin *in vitro*, even potentially at lower concentrations than species that are clinically susceptible to these drugs, such as *Aspergillus* species (Maligie and Selitrennikoff 2005). Based on these data, caspofungin could be expected to be an effective inhibitor of *C. neoformans* growth.

Given these observations, several investigators have tried to explain the discrepancy between the high sensitivity of the target enzyme’s activity and the high tolerance of the organism to caspofungin. Recent work using a fluorescently tagged form of caspofungin has suggested that intracellular concentrations of caspofungin are low in wild-type *C. neoformans* (Huang *et al.* 2016). Therefore, the cell wall or the polysaccharide capsule could prevent the accessibility of caspofungin, a high-molecular weight drug, to its target enzyme. Alternatively, caspofungin could be entering the cell but be rapidly eliminated by degradation or through the action of a multidrug resistance pump. We hypothesized that interrupting specific *C. neoformans* cell processes would render this fungus more susceptible to caspofungin, even at these relatively low effective concentrations. To this end, we screened through two targeted gene deletion collections to identify *C. neoformans* mutants that were hypersensitive to caspofungin relative to the wild-type strain. We also demonstrate possible mechanisms for how the cellular processes controlled by these genes might affect echinocandin resistance in *C. neoformans*.

## Materials and Methods

### Strains, media, and growth conditions

The collections used for the caspofungin sensitivity screen consist of both the 2008 Madhani and 2015 Madhani plate collections, which were purchased from the Fungal Genetics Stock Center (Liu *et al.* 2008; Chun and Madhani 2010). The wild-type (WT) strain used in this study is the clinical strain H99 (Perfect *et al.* 1980). Newly generated strains, as well as strains from alternate sources, are listed in Supplemental Material, Table S1. A similar screen using a subset of these mutant strains was also recently performed (Huang *et al.* 2016).

Strains were maintained on yeast extract, peptone, and dextrose (YPD) agar (10% yeast extract, 20% peptone, 2% dextrose, and 20% Bacto agar), and overnight cultures were incubated in YPD liquid medium. Drug susceptibility testing was performed in yeast nitrogen base medium (1× YNB + 2% glucose) (Pfaller *et al.* 1990; Jessup *et al.* 1998). Cultures for microscopy were prepared in synthetic complete (SC) medium (1× YNB + 1× complete amino acids + 2% glucose).

### Strain creation

To generate the new and independent mutant strains used in this study, targeted gene deletion constructs were designed to replace the entire open reading frame with the neomycin (NEO) or nourseothricin (NAT) dominant selectable markers. Each knockout construct was generated using PCR overlap-extension and a split selectable marker as described previously (Davidson *et al.* 2002; Kim *et al.* 2012). All constructs were transformed into the *C. neoformans* H99 strain by biolistic transformation, as previously described (Toffaletti *et al.* 1993). All deletion primers used in this study can be found in Table S2A.

Upon transformation, strains were selected on YPD medium containing either NAT or NEO. Deletion mutants were checked by a combination of positive and negative confirmation PCRs demonstrating replacement of the WT locus with the mutant allele, followed by Southern blotting to confirm single integration of the deletion constructs (data not shown).

Plasmids used in this study can be found in Table S3. Cloning primers can be found in Table S2B. The overexpressed *GFP-RHO1* construct was generated by cloning the *RHO1* gene plus its native terminator into the *Bam*HI site of the pCN19 vector, which contains the histone H3 promoter and GFP with the *NAT* selection marker. The endogenous *FKS1-GFP* construct was engineered by cloning the following into the pUC19 vector: the *FKS1* gene (without promoter), the *GFP* gene, the *FKS1* terminator, and the *NEO* marker flanked by genomic sequences to target this construct to the *FKS1* locus. These plasmids were transformed as described, and selected on NEO or NAT, and transformants were confirmed by a positive PCR to document the presence of the introduced allele. In the case of the *eFKS1-GFP* construct, PCRs to confirm integration of the construct into the endogenous *FKS1* locus were performed.

### Caspofungin sensitivity primary screen

A small pilot screen revealed that the calcineurin B subunit mutant was hypersensitive to caspofungin compared to the WT, agreeing with published data (Del Poeta *et al.* 2000). This strain became the standard against which to measure other potentially caspofungin-susceptible strains. To determine the optimal conditions under which to perform the caspofungin sensitivity screen, we assessed WT *vs.* *cnb1*Δ growth in YNB medium at caspofungin concentrations between 5 and 50 µg/ml with shaking at 150 rpm at 30° (Cancidas; Merck). We identified 15 μg/ml caspofungin to be a concentration at which the *cnb1*Δ mutant strain was markedly impaired for growth and the WT grew robustly. We then screened the strain collections of 3880 isolates, first preincubating in YPD liquid medium for 16 hr with shaking (150 rpm) at 30°. Cultures were diluted 1:10 in 96-well plates containing either YNB or YNB + caspofungin (15 μg/ml). Strains were incubated with shaking (150 rpm) at 30° for 24 hr, and growth was assessed by measuring OD_600_ on a FLUOStar Optima plate reader (BMG Labtech). Plates were also pin-replicated to YPD plates to assess strain viability after incubation with caspofungin.

After screening, strains were divided into four groups based on caspofungin susceptibility: (1) strains that were inviable postcaspofungin treatment; (2) strains that were viable after caspofungin treatment, but that had significantly decreased growth (OD_600_ that was >2 SD less than the average WT OD_600_); (3) strains that did not have significantly different growth from WT in caspofungin medium; and (4) strains that did not grow in YNB.

### Disc-diffusion secondary screen

To confirm the caspofungin sensitivity of the mutants identified in the above screen, each mutant was incubated overnight in 150 µl of YPD in a 96-well plate with shaking, along with the WT and *cna1*Δ mutant strains as controls. Strains were diluted 1:200 in PBS, then 75 µl per strain was spread onto YNB agar in 6-well plates. Sterile filter discs were placed in the center of each well and 5 μl of 7.5 mg/ml caspofungin was added to each disc. Plates were incubated at 30° for 3 days. After incubation, plates were imaged and zones of inhibition were measured. Mutant phenotypes were classified as WT-like, *cna1*Δ-like, or intermediate.

### Minimal inhibitory concentration assay

Minimal inhibitory concentration (MIC) assays were performed according to modified Clinical Laboratory Standards Institute standard methods for broth microdilution testing of antifungal susceptibility (Pfaller *et al.* 1990; Clinical and Laboratory Standards Institute 2008). In brief, cells were diluted in phosphate-buffered saline (PBS) to an OD_600_ of 0.25, then diluted 1:100 in YNB medium. Caspofungin was diluted in PBS. Next, 2× working stocks of caspofungin were prepared in YNB medium, then caspofungin was serially diluted twofold in 100 μl YNB medium, following which 100 µl of 1:100 dilution of cells was added to diluted drug in 96-well plates. The final concentration range of caspofungin was 200–0.39 µg/ml. Plates were incubated for 48 hr without shaking at 30° or 35°. After 48 hr, OD_600_ was measured on a FLUOStar Optima plate reader. MIC_50_ values were calculated by calculating relative growth using (drug-treated OD_600_/untreated OD_600_), with MIC_50_ corresponding to a ≥ 50% decrease in relative growth.

### Checkerboard assay

Checkerboard assays to assess antifungal drug synergy using the fractional inhibitory concentration index (FIC) for combinations of compounds were performed as described (National Committee for Clinical Laboratory Standards 1992; Franzot and Casadevall 1997). In brief, the WT strain H99 was inoculated from plated colonies into PBS at an OD_600_ of 0.25, and subsequently diluted 1:100 into RPMI medium. Nikkomycin Z stock was diluted in PBS (nikkomycin Z; Sigma [Sigma Chemical], St. Louis, MO). Manumycin A, clorgyline, and 2-bromopalmitate (2BP) were diluted in DMSO (manumycin A, Bioviotica; clorgyline, Sigma; and 2BP, Sigma). Caspofungin was diluted for a final concentration range between 100 and 1.5625 μg/ml, and the test drugs were diluted to the following final concentration ranges: nikkomycin Z 400 to 0.78125 μg/ml, manumycin A 40 to 0.078 μM, 2BP 400 to 0.78125 µM, and clorgyline 100 to 1.5625 µM. Assays were incubated at 30 and 37°. FIC index values for a combination of compounds A and B were calculated as:

$$\text{FIC}=\frac{MIC_{A}\;in\;combination}{MIC_{A}\;alone}+\frac{MIC_{B}\;in\;combination}{MIC_{B}\;alone}$$

where an FIC index value of < 1.0 is considered synergistic (with < 0.5 considered strongly synergistic), additive if the value was 1.0, autonomous if the value was between 1.0 and 2.0, and antagonistic if the FIC index was > 2.0 (Franzot and Casadevall 1997).

### Whole-genome sequencing, alignment, and variant calling

Whole-genome sequencing was performed on both the WT background strain and the *msh1*Δ mutant strain, by the Duke Center for Genome and Computational Biology Genome Sequencing Shared Resource using an Illumina MiSeq instrument. Paired-end libraries were sequenced with read lengths of 251 bases. Reads were aligned to the version 3 H99 genome (Janbon *et al.* 2014) using BWA-MEM with default settings (Li and Durbin 2009). The Genome Analysis Toolkit (GATK) best practices pipeline (McKenna *et al.* 2010) was used in combination with SAMtools (Li *et al.* 2009) and Picard to realign reads before SNP calling using the UnifiedGenotyper component of GATK with the haploid ploidy setting. The resulting variant call formats were filtered using VCFtools (Danecek *et al.* 2011) and annotated for variant effect using SnpEff (Cingolani *et al.* 2012). Heterozygous calls were removed as presumed mismapped repetitive regions. Raw reads are available on the National Center for Biotechnology Information (NCBI) Sequence Read Archive under accession number PRJNA501913.

### Chitin and chitosan assay

The chitin and chitosan contents of *C. neoformans* cell walls were assessed as described (Banks *et al.* 2005). Briefly, cells were incubated overnight in YPD. Cultured cells were then diluted to an OD of 0.8 in SC or SC + 15 μg/ml caspofungin, and incubated with shaking at 30° for 6 hr. Cells were divided and lyophilized, then either mock treated or treated with acetic anhydride to acetylate chitosan to form chitin. Cell walls were then digested with 5 mg/ml chitinase for 72 hr. Monomer levels of *N*-acetylglucosamine were assessed by a *p*-dimethylaminobenzaldehyde colorimetric assay and read on a FLUOStar Optima plate reader. Acetic anhydride samples represented levels of both chitin and chitosan in the cell wall, while untreated samples represented chitin alone. Chitosan levels were calculated as the difference between the acetic anhydride-treated and the untreated samples. Data were analyzed using a two-way ANOVA, followed by Student’s *t*-tests to determine statistical significance.

### Cell wall staining and microscopy

Cells were prepared for cell wall staining as described (Ost *et al.* 2017). WT cells were cultured overnight in YPD medium. Overnight cultures were diluted to an OD_600_ of 1 in 15 ml SC or SC plus caspofungin (5, 10, or 20 µg/ml caspofungin), and incubated with shaking at 30°. At the indicated timepoints, 1 ml aliquots of each culture were collected and stained with calcofluor white (CFW). Cells were pelleted at 5000 rpm for 2 min, then resuspended in 100 µl PBS + 25 μg/ml CFW and incubated in the dark at room temperature for 10 min. Cells were then washed two times with PBS and resuspended in 50 μl PBS for imaging. Strains were imaged on a Zeiss ([Carl Zeiss], Thornwood, NY) Axio Imager A1 fluorescence microscope equipped with an Axio-Cam MRM digital camera to capture both DIC and fluorescent images. Cell wall-staining fluorescence intensity was analyzed using Fiji software, and the mean gray values were analyzed (Schindelin *et al.* 2012). Data presented represent the average fluorescence values. Data were analyzed using a two-way ANOVA, followed by Student’s *t*-tests to determine statistical significance.

For fluorescent fusion protein microscopy, strains were incubated in SC medium for 18 hr at 30° with shaking. These cultures were pelleted at 3000 rpm for 5 min, and resuspended in SC or SC + 15 μg/ml caspofungin. Strains were incubated with shaking at 30° for 90 min, with aliquots collected for imaging at 15-min intervals. Aliquots were incubated with NucBlue Live Ready Probes reagent for 5 min, pelleted at 5000 rpm for 2 min, then resuspended in 50 μl SC (Thermo Fisher Scientific). Strains were imaged on a Zeiss Axio Imager A1 fluorescence microscope equipped with an Axio-Cam MRM digital camera to capture both DIC and fluorescent images. For the Fks1-GFP and GFP-Rho1 localization experiments, cultures were incubated for 18 hr at 30° with shaking in SC medium. These cultures were normalized to an OD_600_ of 2.0 in SC plus 0, 5, 10, or 15 μg/ml caspofungin, and imaged at 20-min intervals for 1.5 hr.

### RNA preparation and quantitative real-time PCR

The WT strain was grown in YPD for 18 hr at 30° with shaking. Cells were then inoculated at an OD_600_ of 1.5 into 5 ml SC, SC + 10 µg/ml caspofungin, or SC + 15 μg/ml caspofungin. Cultures were incubated at 30° with shaking for 90 min, then cells were harvested by centrifugation at 3000 rpm for 5 min and lyophilized. RNA was isolated using a RNeasy Plant Mini Kit (QIAGEN, Valencia, CA), with the addition of bead beating for 1 min prior to lysis and on-column DNase treatment (QIAGEN). cDNA was prepared using the AffinityScript QPCR cDNA synthesis kit using oligo-dT primers to bias for mRNA transcripts (Agilent Genomics). Quantitative real-time PCR was performed using PowerUp SYBR Green Master mix (Applied Biosystems, Foster City, CA) on a QuantStudio 6 Flex system. Real-time PCR primers are listed in Table S2C (Esher *et al.* 2018). Data were analyzed using a two-way ANOVA, followed by Student’s *t*-tests to determine statistical significance.

### Data availability

Strains and plasmids are available upon request. File S1 contains a list and descriptions of all supplemental files. Sequence data are available at the NCBI Sequence Read Archive under the accession number PRJNA501913. Supplemental material available at FigShare: https://doi.org/10.25386/genetics.8085290

## Results

### Initial screen for processes contributing to caspofungin tolerance in *C. neoformans*

We performed a forward genetic screen of targeted deletion mutants to identify cellular processes that contribute to *C. neoformans* tolerance to caspofungin treatment. Using two screening methods in sequence, we screened 3880 mutants for altered growth during caspofungin treatment at 30° (Liu *et al.* 2008; Chun and Madhani 2010). We specifically chose this permissive incubation temperature for our initial screens to potentially capture pathways that may be required for thermotolerance, and for which mutant strains would therefore not be viable at higher temperatures. Our screening methods yielded 54 mutants that appeared to have reduced caspofungin tolerance relative to the WT strain. We then performed broth microdilution assays to compare caspofungin susceptibility to the WT strain, as well as to a *cna1*Δ strain with a mutation in the calcineurin A subunit gene. Strains with altered calcineurin function are known to be more susceptible to caspofungin (Del Poeta *et al.* 2000; Kraus *et al.* 2003). Of the mutants identified in the primary screen, 14 were confirmed to have caspofungin susceptibility similar to or greater than the *cna1*Δ mutant strain (Table 1). This strict screening criterion enabled us to focus our studies exclusively on more highly drug-susceptible mutants (Table 1). The remaining strains displayed only minimal increases in sensitivity to caspofungin and they were not tested further. Of note, we identified the *cnb1*Δ calcineurin regulatory subunit mutant, which phenocopies the *cna1*Δ mutant, as a caspofungin-hypersensitive mutant in our screen, validating our screening methods (Odom *et al.* 1997; Del Poeta *et al.* 2000; Fox *et al.* 2001; Kraus *et al.* 2003).

**Table 1:** Caspofungin MICs for screen hits

Gene locus tag	Gene product mutated	MIC (µg/ml)
CNAG_00375	SAGA complex histone acetyltransferase (Gcn5)	12.5
CNAG_02682	Hypothetical protein (Msh1 homolog)	0.78
CNAG_05070	Sulfite reductase (NADPH) hemoprotein, β-component	0.78
CNAG_06902	Hypothetical protein	12.5
CNAG_02236	Type 2A-like serine/threonine protein phosphatase (Ppg1)	0.78
CNAG_03981	Palmitoyltransferase Pfa4	6.25
CNAG_03841	Hypothetical protein	12.5
CNAG_02292	Copper chaperone Lys7	12.5
CNAG_04992	Hypothetical protein	12.5
CNAG_03080	Fatty acid elongase	12.5
CNAG_07636	Chitin synthase regulator (Csr2)	3.125
CNAG_02891	Endoplasmic reticulum rhodanese-like protein (Rdl2)	12.5
CNAG_01717	Cell differentiation protein Rcd1	12.5
CNAG_00888	Calcineurin B subunit	3.125

MIC, minimal inhibitory concentration. SAGA: Spt3-Ada2-Gcn5 complex.

Within the list of sensitive mutants, we identified multiple biological processes that seem to be important for caspofungin tolerance based on gene ontology (GO) analysis (see Table S4 for full GO analysis) (Stajich *et al.* 2012). Of note, the *msh1*Δ mutant strain displayed a very low caspofungin MIC. This strain has a defect in a predicted homolog of a MutS mismatch repair protein most likely functioning on mitochondrial DNA (Sia and Kirkpatrick 2005). Interestingly, independently created *C. neoformans msh1*Δ mutants did not display the caspofungin-susceptible phenotype of the original mutant, suggesting that secondary mutations in this strain were the cause of increased drug susceptibility. Indeed, whole-genome sequencing of the original *msh1*Δ mutant strain revealed several additional mutations in both the nuclear and mitochondrial genomes, as predicted for a strain with potential defects in DNA repair mechanisms (Table S5).

### Calcineurin signaling plays a role in caspofungin tolerance in *C. neoformans*

In previous *in vitro* studies, the calcineurin inhibitor FK506 has demonstrated synergistic interactions with caspofungin against *C. neoformans* (Del Poeta *et al.* 2000). In our caspofungin sensitivity screen, we identified a mutant of the calcineurin B regulatory subunit, *cnb1*Δ, to be highly sensitive to caspofungin, as seen in previous work (Kraus *et al.* 2003). Indeed, mutants of both the calcineurin A catalytic and B regulatory subunits—*cna1*Δ and *cnb1*Δ, respectively—exhibit an eightfold increase in caspofungin sensitivity when compared to the WT strain (Table 1 and Table 2). Therefore, we strove to identify the mechanism by which this phosphatase is facilitating caspofungin tolerance in *C. neoformans* by examining the activation of a known target of calcineurin signaling in *C. neoformans*, the Crz1 transcription factor.

**Table 2:** Additional caspofungin MICs at 30°

Gene locus tag	Gene product mutated	MIC (µg/ml)
WT	NA	25
CNAG_04796	Calcineurin A subunit	3.125
CNAG_01744	Crz1 transcription factor	12.5
CNAG_05581	Chitin synthase 3	3.125
CNAG_00293	Ras1 GTPase	25
CNAG_05348	Cdc42 GTPase	25
CNAG_05968	Cdc420 GTPase	25
CNAG_04243	Cdc24 guanine nucleotide exchange factor	25
CNAG_06165	Ste20 PAK kinase	25
CNAG_04761	Ras2 GTPase	25
CNAG_05998	Rac2 GTPase	25
CNAG_02883	Rac1 GTPase	25
CNAG_05925	Septin Cdc3	25
CNAG_01740	Septin Cdc12	25
CNAG_05970	PAK kinase Pak1	25

MIC, minimal inhibitory concentration; WT, wild-type. PAK: p21-activated protein kinase.

**Figure 1:**
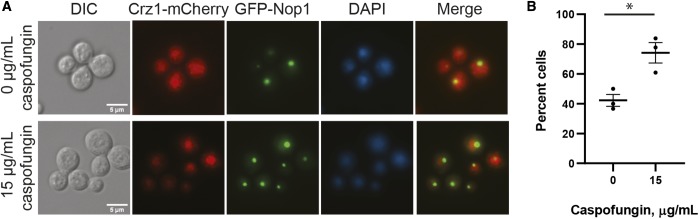
Crz1 is activated and localizes to the nucleus in response to caspofungin treatment. (A) A strain expressing Crz1-mCherry and GFP-Nop1 (nucleolar marker) was incubated in SC medium with either 0 or 15 µg/ml caspofungin for 45 min, then stained with NucBlue Live Cell nuclear stain for 5 min and imaged on a Zeiss AxioVision epifluorescence microscope. Bar, 5 μm. (B) Percentage of cells displaying nuclear Crz1-mCherry localization as measured by colocalization with GFP-Nop1. Results represent percentage nuclear localization from three independent experiments. Error bars represent SEM. * *P*-value < 0.05.

The Crz1 transcription factor is activated through dephosphorylation by calcineurin, and Crz1 mediates many of the known transcriptional effects of this calcineurin signaling (Lev *et al.* 2012). We assessed the effect of caspofungin on Crz1 nuclear localization by examining mCherry-tagged Crz1 fusion protein (Crz1-mCherry) colocalization with the GFP-Nop1 nucleolar marker, as well as with DAPI staining (Chow *et al.* 2017). In untreated cells, Crz1-mCherry remains localized in the cytosol. We determined that Crz1-mCherry localizes to the nucleus after 45 min of incubation in 15 µg/ml of caspofungin (Figure 1, A and B). The nuclear localization of Crz1-mCherry suggests that Crz1 is likely being activated under these conditions. However, the *crz1*Δ mutant displayed a caspofungin MIC more similar to WT than to the *cna1*Δ or *cnb1*Δ mutant strains (Table 2). Together, these results suggest that, although Crz1 is being activated during caspofungin treatment, caspofungin tolerance is mediated in part through calcineurin-dependent but Crz1-independent targets. Therefore, we assessed the caspofungin susceptibility of several strains with loss-of-function mutations in downstream calcineurin targets (Table S6) (Park *et al.* 2016). Unlike the *cna1*Δ or *cnb1*Δ calcineurin subunit mutants, none of the tested strains deficient in individual downstream calcineurin effector genes displayed altered caspofungin susceptibility. These data are consistent with emerging reports that *C. neoformans* calcineurin acts through both Crz1-dependent and -independent processes (Lev *et al.* 2012; Chow *et al.* 2017). Alternatively, there may be functional redundancy among the calcineurin effectors with respect to caspofungin susceptibility.

### The Pfa4 palmitoyltransferase plays a role in caspofungin tolerance through regulation of its target proteins

The Pfa4 palmitoyltransferase is required for the addition of palmitoyl groups to various *C. neoformans* proteins (Nichols *et al.* 2015; Santiago-Tirado *et al.* 2015). This post-translational modification is necessary for the proper localization and function of these target proteins. In our MIC assays, we documented a fourfold increase in caspofungin susceptibility of the *pfa4*∆ strain at 30°. Given the temperature sensitivity of this mutant strain (Nichols *et al.* 2015; Santiago-Tirado *et al.* 2015), we would not have isolated it in screens at higher incubation temperatures. The caspofungin sensitivity of the *pfa4*∆ mutant was more pronounced at 35°, a temperature at which the *pfa4*Δ mutant grows but with a modest growth delay compared to WT (Table 1 and Table 3) (Nichols *et al.* 2015; Santiago-Tirado *et al.* 2015).

**Table 3:** Additional caspofungin MICs at 35°

Gene locus tag	Gene product mutated	MIC (µg/ml)
H99	N/A	25
CNAG_00293	Ras1 GTPase	6.25
CNAG_05348	Cdc42 GTPase	12.5
CNAG_05968	Cdc420 GTPase	25
CNAG_04243	Cdc24 guanine nucleotide exchange factor	12.5
CNAG_06165	Ste20 PAK kinase	25
CNAG_04761	Ras2 GTPase	25
CNAG_05998	Rac2 GTPase	25
CNAG_02883	Rac1 GTPase	25
CNAG_05925	Septin Cdc3	12.5
CNAG_01740	Septin Cdc12	6.25
CNAG_05970	PAK kinase Pak1	25
CNAG_03981	Palmitoyltransferase Pfa4	0.39

MIC, minimal inhibitory concentration.

Additionally, since Pfa4-mediated palmitoylation also seems to play a role in caspofungin tolerance in *C. neoformans*, we assessed whether there might be synergy between caspofungin and inhibitors of palmitoyltransferases. 2BP is a competitive inhibitor of palmitoyltransferases with antifungal activity against *Aspergillus fumigatus* (Jennings *et al.* 2008; Fortwendel *et al.* 2012). In contrast, we found that 2BP displayed limited antifungal activity alone against *C. neoformans*, with an MIC of 25–50 µM. However, 2BP displayed synergy with caspofungin at both 30 and 37° (Table 4). This observed pharmacologic synergy between caspofungin and 2BP is consistent with the synthetic effect on growth inhibition between caspofungin and the *pfa4*Δ mutation.

**Table 4:** FIC indices in combination with caspofungin

Drug tested in combination	Combination drug MIC	FIC index (30°)	Drug relationship	Combination drug MIC	FIC index, (37°)	Drug relationship
Manumycin A	5 µM	0.508–0.75	Synergistic	1.25 µM	0.562–0.75	Synergistic
2BP	25–50 µM	0.266–0.625	Synergistic to strongly synergistic	12.5 µM	0.188–0.531	Synergistic to strongly synergistic
Nikkomycin Z	100 µg/ml	1	Additive	ND	ND	ND
Clorgyline	25–50 µM	0.25–0.281	Strongly synergistic	25–50 µM	0.18–0.625	Synergistic to strongly synergistic

FIC, fractional inhibitory concentration; MIC, minimal inhibitory concentration; ND, not determined.

Since Pfa4 is responsible for the regulation of various functions within the cell, it is likely that this caspofungin sensitivity is due to dysregulation of one or more Pfa4 palmitoylation targets. Moreover, the additional growth and drug-sensitivity phenotypes at 35° are in concordance with known targets of Pfa4 being required for full thermotolerance in *C. neoformans* (Nichols *et al.* 2015). Therefore, the caspofungin susceptibility caused by mutations in several Pfa4-regulated gene products was assessed.

### The thermotolerance arm of the Ras signaling pathway is required for caspofungin tolerance

**Figure 2:**
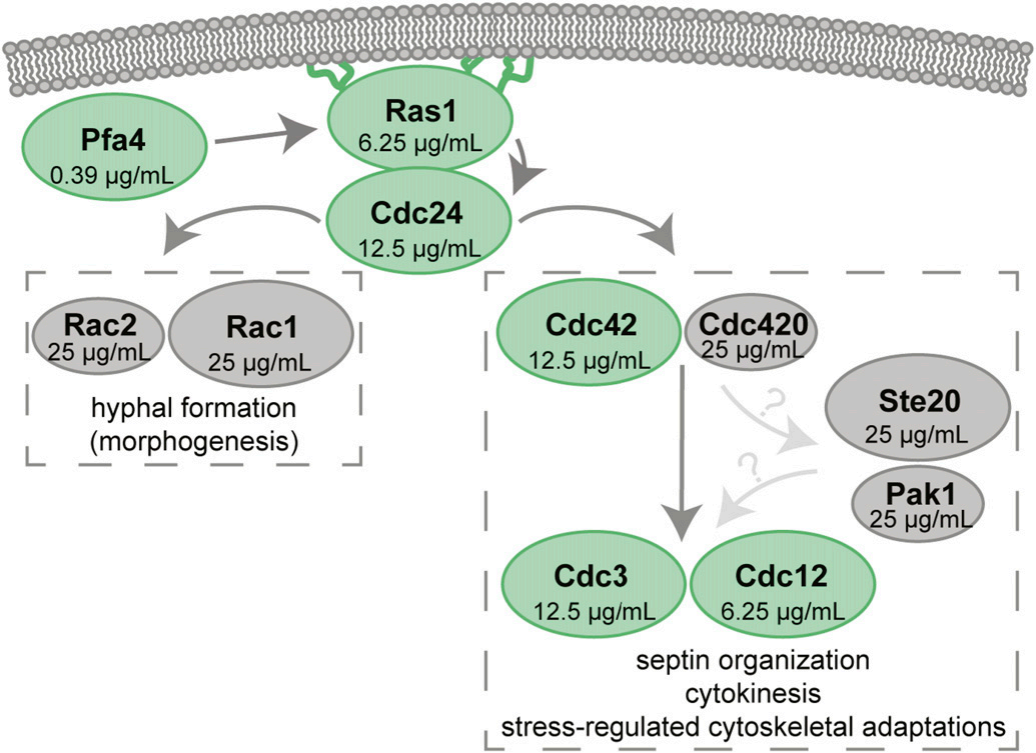
Ras1 signaling partner mutants are differentially affected by caspofungin treatment. Model of Ras signaling in *C. neoformans*. Paired paralogs are represented as the major (larger oval) and minor (smaller oval) paralog (Ballou *et al.* 2013a). Caspofungin minimal inhibitory concentrations at 35° for each component are presented within each oval. The green ovals represent mutants that displayed increased caspofungin susceptibility, while the gray ovals display wild-type caspofungin susceptibility.

Pfa4 palmitoylates the *C. neoformans* Ras1 GTPase, and this post-translational modification is required for the proper subcellular localization of Ras1, as well as its function (Nichols *et al.* 2015). To determine whether the caspofungin susceptibility of the *pfa4*Δ mutant is reflected in this downstream target pathway, we assessed caspofungin susceptibility of the *ras1*Δ mutant, as well as for mutants in the Ras1 morphogenesis (mediated by Rac proteins) and Ras1 thermotolerance subpathways (mediated by Cdc24/Cdc42 and the septin proteins) (Figure 2) (Waugh *et al.* 2002; Nichols *et al.* 2007; Ballou *et al.* 2009, 2013a,b). The *ras1*∆ mutant is fourfold more sensitive to caspofungin than WT. Similar increases in caspofungin susceptibility were also noted for the *cdc42*Δ and *cdc24*Δ mutants, as well as the *cdc3*Δ and *cdc12*Δ septin mutants, which are further downstream effectors of the *C. neoformans* Ras thermotolerance pathway (Nichols *et al.* 2007; Ballou *et al.* 2009, 2013a). These proteins mediate dynamic actin cytoskeletal changes associated with budding and cell division, and a robust septin complex is required for *C. neoformans* to grow in the presence of cell stresses such as elevated temperature. Therefore, each of these signaling proteins is required for cryptococcal viability at elevated temperatures and other states of cell stress. Accordingly, the changes in caspofungin susceptibility in the corresponding mutant strains were only observed at 35°, not 30°, indicating that the elements of this pathway are necessary for caspofungin tolerance under conditions at which the pathway is activated (Table 2 and Table 3). In contrast, the Rac1 and Rac2 proteins are not required for caspofungin tolerance. These Ras1-mediated GTPases are involved in a distinct signaling pathway controlling morphological transitions, such as hyphal formation during mating (Ballou *et al.* 2013b).

Although specific inhibition of fungal Ras activity is limited by the highly conserved nature of this protein, recent investigations have explored inhibitors of Ras-modifying enzymes as antifungal agents (Hast *et al.* 2011; Selvig *et al.* 2013; Esher *et al.* 2016; Pianalto and Alspaugh 2016). In addition to palmitoylation, Ras-like GTPases also require the post-translational addition of lipophilic prenyl groups to C-terminal cysteine residues for their proper localization and function. These lipid additions are catalyzed by protein-farnesyltransferases and/or protein geranylgeranyltransferases (Palsuledesai and Distefano 2014). Additionally, protein-farnesyltransferase inhibitors (FTIs) could be affecting the function of not only Ras proteins, but other farnesylated proteins. Therefore, we assessed the pharmacological synergy between caspofungin and the FTI manumycin A (Hast *et al.* 2011). Manumycin A treatment has been demonstrated previously to disrupt the plasma membrane localization of GFP-Ras1 in *C. neoformans*, consistent with its biochemical activity as an FTI (Hast *et al.* 2011). In checkerboard MIC assays, manumycin A displayed synergy with caspofungin, with FIC indices ranging from 0.508 to 0.75 at 30°, and from 0.562 to 0.75 at 37° (Table 4) (Franzot and Casadevall 1997). Given the limited intrinsic antifungal activity of this first-generation FTI, these results suggest that Ras inhibitors with greater anticryptococcal activity might be promising coadministered agents to augment the effect of caspofungin against *C. neoformans*.

### Chitin synthesis and the Chs3 chitin synthase are involved in the response to caspofungin treatment

**Figure 3:**
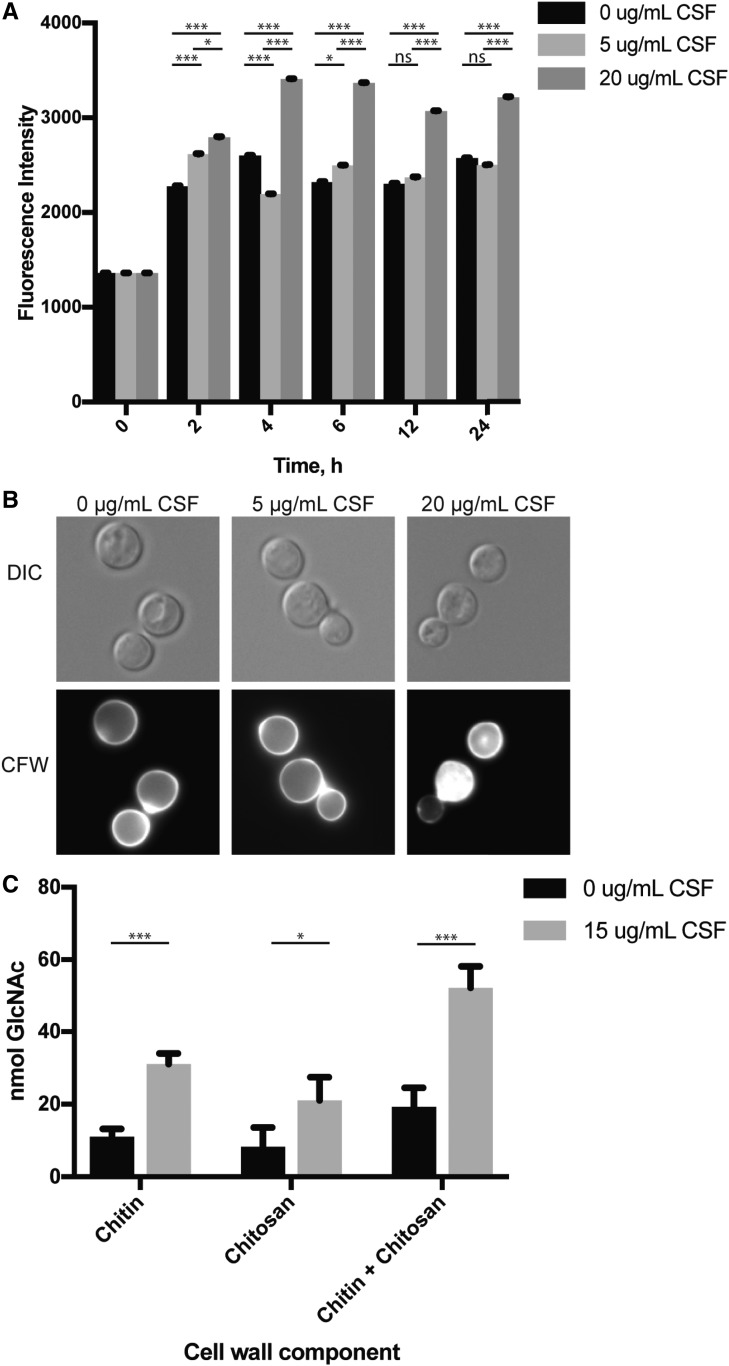
Cell wall chitin and chitosan levels increase during CSF treatment. (A) Quantification of CFW staining of *C. neoformans* wild-type cells treated with CSF. Cells were incubated in SC medium at 30° with 0, 5, or 20 µg/ml CSF over 24 hr. At each timepoint, cells were harvested and stained with CFW to assess total cell wall chito-oligomer content, then imaged on a Zeiss AxioVision epifluorescence microscope. Images were masked for fluorescence and average fluorescent intensity for each cell was quantified using ImageJ. Each timepoint represents quantification of > 80 cells over at least three images. Error bars represent SEM. Statistics performed were two-way ANOVA, followed by pair-wise Student’s *t*-tests. * *P* < 0.05 and *** *P* < 0.001. (B) Representative images of cells treated as above with 0, 5, or 20 µg/ml CSF, and then stained for chitin content with CFW at 4 hr post-CSF treatment. (C) Quantification of cell wall chitin and chitosan using a DMAB colorimetric assay. Cells were incubated in SC ± 15 µg/ml CSF at 30° for 6 hr, then harvested and the assay performed as described. Error bars represent SEM. Statistics performed were two-way ANOVA, followed by pair-wise Student’s *t*-tests. * *P* < 0.05 and *** *P* < 0.001. CFW, calcofluor white; CSF, caspofungin; DMAB, *p*-dimethylaminobenzaldehyde; GlcNAc, *N*-acetylglucosamine; ns, not significant.

One of the most prominent targets of Pfa4 palmitoyltransferase activity is the Chs3 chitin synthase, which is responsible for the biosynthesis of chitin that is destined to become chitosan in the cell wall (Banks *et al.* 2005; Baker *et al.* 2007). In addition to the *pfa4*Δ mutant strain, our screen also identified the chitin synthase regulator mutant strain *csr2*Δ as a highly caspofungin-sensitive strain (Table 1). Csr2 and Chs3 coregulate chitin synthesis in *C. neoformans* (Banks *et al.* 2005). Given the altered caspofungin tolerance of two mutants predicted to affect the function of the Chs3 chitin synthase, we assessed the caspofungin susceptibility of a *chs3*∆ strain to determine if misregulation of this protein, and a lack of proper chitin and chitosan deposition, might contribute to the caspofungin susceptibility of the *pfa4*Δ mutant strain. The *chs3*Δ mutant strain was eightfold more sensitive to caspofungin than the WT strain (Table 2). These results are consistent with observations in *A. fumigatus* and *Candida* species in which compensatory increases in cell wall chitin are induced upon exposure of these fungi to caspofungin (Walker *et al.* 2008, 2012; Fortwendel *et al.* 2010; Verwer *et al.* 2012). To further explore the interaction of caspofungin treatment and a response involving chitin biosynthesis in the cell wall, we used the chito-oligomer-binding dye CFW to assess changes in *C. neoformans* cell wall chitin content in response to caspofungin treatment. We observed a dose-dependent increase in CFW staining for cells treated with caspofungin compared to untreated cells, with the most significant difference being at the highest concentration of caspofungin (Figure 3, A and B). Accordingly, when we assessed cell wall chitin and chitosan composition using an *in vitro* colorimetric assay (Banks *et al.* 2005), we found that *C. neoformans* cells treated with 15 µg/ml caspofungin have an ∼2.5-fold increase in both chitin and chitosan compared to untreated cells (Figure 3C).

Since the *chs3*Δ chitin synthase mutant strain is so susceptible to the effects of caspofungin, we hypothesized that the coadministration of caspofungin with the chitin synthase inhibitor nikkomycin Z might result in a similar synergistic antifungal effect. We tested antifungal drug interactions using a checkerboard MIC assay, using varying concentrations of each drug in combination. In this assay, we did not observe synergy between caspofungin and nikkomycin Z, one of the few known inhibitors of chitin synthesis in some fungi (Gaughran *et al.* 1994; Li and Rinaldi 1999). The FIC index, a marker of drug interaction, was 1.0, suggesting an additive effect between the two compounds rather than drug synergy (Table 4). However, historically, nikkomycin Z has not been an effective inhibitor of *C. neoformans* growth, potentially due to the redundancy in chitin synthase genes in this organism (Li and Rinaldi 1999; Kraus *et al.* 2003; Banks *et al.* 2005). Eight chitin synthases have been identified in *C. neoformans*, with both distinct and overlapping functions (Banks *et al.* 2005). While the specific biochemical target of nikkomycin Z has yet to be identified in *C. neoformans*, treatment with nikkomycin Z activates the Pkc1 cell wall integrity pathway, suggesting that nikkomycin Z inhibits cell wall biosynthesis (Kraus *et al.* 2003). Given the very limited antifungal activity of nikkomycin Z against *C. neoformans* and our new genetic studies, we propose that there is potential for synergy between caspofungin and future, more effective chitin synthase inhibitors, or other cell wall biosynthesis inhibitors.

### Cell wall gene expression is altered during caspofungin treatment

**Figure 4:**
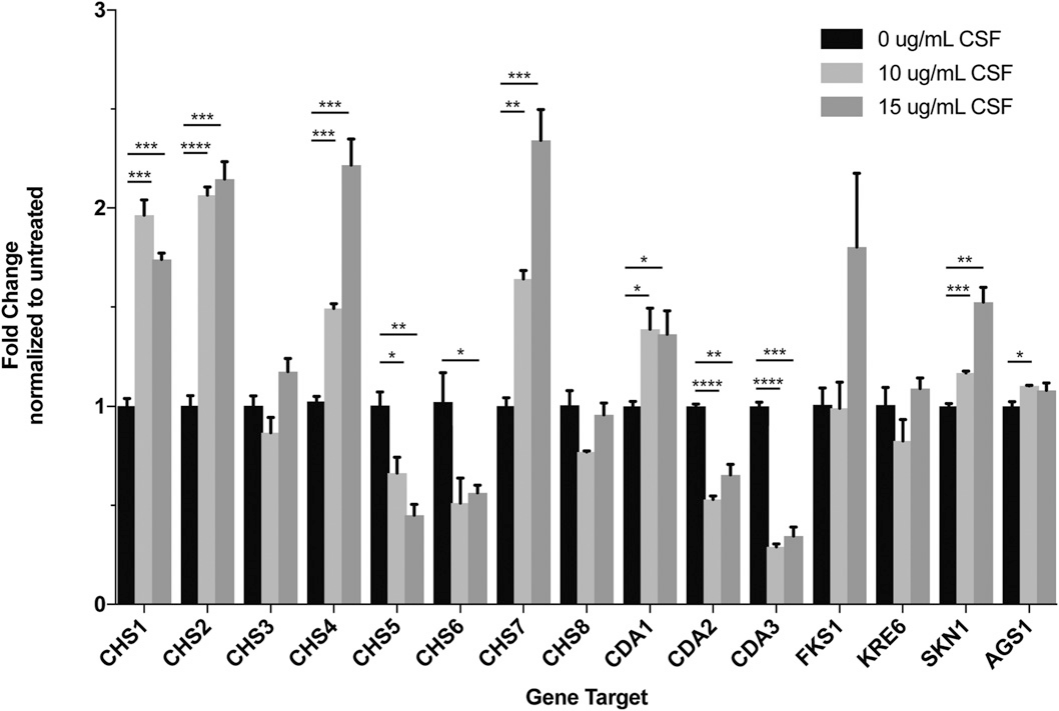
Cell wall biosynthesis gene expression is altered in response to CSF. Wild-type *C. neoformans* cells were incubated in SC medium containing 0, 10, or 15 µg/ml CSF for 90 min, followed by RNA purification and quantitative real-time PCR for the indicated target genes, using the *GPD1* gene as an internal control. The results represent average values for biological triplicate samples. Statistical significance was assessed using two-way ANOVA, followed by Student’s *t*-test for pair-wise comparisons. * *P* < 0.05, ** *P* < 0.01, *** *P* < 0.001, and **** *P* < 0.0001. CSF, caspofungin.

Given the caspofungin-induced changes in cell wall chitin content, we explored whether the expression of cell wall biosynthesis genes was altered in response to caspofungin treatment. We assessed the transcript abundance for genes involved in the synthesis of chitin (*CHS1*, *CHS2*, *CHS3*, *CHS4*, *CHS5*, *CHS6*, *CHS7*, and *CHS8*), chitosan (*CDA1*, *CDA2*, and *CDA3*), α-1,3-glucan (*AGS1*), β-1,3-glucan (*FKS1*), and β-1,6-glucan (*KRE6* and *SKN7*) (Thompson *et al.* 1999; Banks *et al.* 2005; Reese *et al.* 2007; Gilbert *et al.* 2010; Esher *et al.* 2018). Many of these cell wall biosynthesis genes demonstrate altered regulation in response to caspofungin treatment (Figure 4). *CHS1*, *CHS2*, *CHS4*, *CHS7*, *SKN1*, and *CDA1* all exhibited significant increases in expression, especially at the highest concentration of caspofungin tested in this experiment. These results may reflect some of the compensatory processes seen in other fungi, such as *Candida* species and *A. fumigatus*, in which increased cell wall chitin has been proposed to be a mechanism for these fungi to overcome the effects of caspofungin treatment (Walker *et al.* 2008; Fortwendel *et al.* 2010). However, we also noted the potential involvement of more diverse cell wall components. Additionally, the upregulation of *SKN1* suggests a potential role for β-1,6-glucan synthesis in the caspofungin tolerance mechanism for *C. neoformans*. In light of previous data showing that caspofungin exposure results in a decrease in β-1,6-glucan staining via immunoelectron microscopy, there is the potential that the upregulation of β-1,6-glucan biosynthetic genes could represent transcriptional compensation for secondary cell wall effects after drug treatment (Feldmesser *et al.* 2000). Interestingly, *CHS5*, *CHS6*, *CDA2*, and *CDA3* all displayed decreased expression during caspofungin treatment. Therefore, though not all cell wall-associated genes are upregulated in response to caspofungin, there seems to be a decisive alteration in the expression of cell wall biosynthesis and modification genes, reflecting a coordinated compensatory response to the cell wall inhibitor caspofungin.

### Fks1-GFP localization is not altered during caspofungin treatment

Since we did not observe statistically significant differences in *FKS1* transcript levels after exposure to caspofungin, we assessed whether treatment with this drug has a direct effect on the cellular localization of the β-1,3-glucan synthase. We examined the localization of the two components of the complex: the Fks1 catalytic subunit and the Rho1 regulatory subunit, each as a fusion protein tagged with GFP. We treated *C. neoformans* strains expressing either endogenous Fks1-GFP or overexpressed GFP-Rho1 in a gradient of caspofungin concentrations, and assessed localization over 1 hr of treatment (Figure S1). Both Fks1-GFP and GFP-Rho1 localized to endomembranes, including structures that resemble perinuclear endoplasmic reticulum staining (Pianalto *et al.* 2018). Similarly, both proteins also localized to the outer edge of the cell. Fks1-GFP, in particular, appeared to localize to the plasma membrane, but in distinct patches rather than along the entire cell surface. Therefore, there was no significant difference in Fks1-GFP or GFP-Rho1 localization in response to caspofungin treatment.

### Inhibition of efflux pumps increases potency of caspofungin

Recent studies suggest that caspofungin is not efficiently maintained at high intracellular concentrations in *C. neoformans* (Huang *et al.* 2016). Therefore, we hypothesized that this organism might be preventing the intracellular accumulation of caspofungin through the activity of one or more efflux pumps. To address this hypothesis, we performed a drug interaction assay to determine whether caspofungin potency might be enhanced in the presence of clorgyline, a monoamine oxidase inhibitor that also acts as an inhibitor of ATP-binding cassette (ABC) transporters in other fungal species (Holmes *et al.* 2012). This pharmacological approach was pursued since there are > 50 predicted ABC transporters and > 150 predicted major facilitator superfamily proteins encoded in the *C. neoformans* genome (Loftus *et al.* 2005; Holmes *et al.* 2016). We found that the efflux inhibitor clorgyline and caspofungin demonstrated a strong synergistic interaction, with an FIC index ranging from 0.25 to 0.281 at 30° and 0.18–0.625 at 37° (Table 4). These data suggest that drug efflux might be a contributor to the innate tolerance of *C. neoformans* to caspofungin treatment.

## Discussion

Despite the medical importance of fungal infections, few novel antifungal drugs have been introduced in recent years. Echinocandins, one of the most recently approved classes of antifungal drugs, were especially promising given their limited toxicity profile and novel mechanism of action. However, these agents display limited efficacy against several fungal classes, including the thermally dimorphic fungi and *Cryptococcus* species (Abruzzo *et al.* 1997; Bartizal *et al.* 1997; Espinel-Ingroff 1998).

Our screen, using a collection of *C. neoformans* mutant strains, revealed that cryptococcal tolerance to the echinocandin drug caspofungin is likely a multifaceted phenomenon. We identified several pathways and processes important for full tolerance of caspofungin, including those associated with cell surface integrity, such as chitin biosynthesis and the phosphatase Ppg1, or general stress tolerance, such as calcineurin. Indeed, one of the most caspofungin-sensitive mutants that we identified was a *ppg1*Δ mutant. Ppg1 is a phosphatase that has been shown to be necessary for full cell wall integrity, even under nonstress conditions, but that is also required for survival in cell wall stress conditions (Gerik *et al.* 2005). However, Ppg1 is not a part of the traditional cell wall integrity MAP kinase pathway in *C. neoformans*. Interestingly, we did not see hypersensitivity among mutants in the cell wall integrity MAPK pathway, which suggests that noncanonical cell wall integrity signaling could be playing a role in the response to caspofungin-induced cell wall stress (Gerik *et al.* 2005). We also found multiple proteins that have potential antioxidant roles, such as an Rdl2 Rhodanese homolog and a putative Lys7 homolog, which typically partners with superoxide dismutase 1 (Culotta *et al.* 1997; Orozco *et al.* 2012). We chose to focus our initial evaluation on strains with mutations in cellular pathways previously implicated in *C. neoformans* pathogenesis.

Another recent study assaying *C. neoformans* mutants for caspofungin sensitivity identified several other processes that were not identified in this study. The differences in the caspofungin-susceptible *C. neoformans* mutants identified in the two different studies are likely explained by variations in experimental conditions. First, the two screens used overlapping but different mutant libraries for the screens, as well as different cutoffs for defining altered caspofungin susceptibility. Also, we specifically chose to perform our screens at 30° to identify mutants that might have thermotolerance defects; for example, mutants such as the calcineurin subunit *cnb1*Δ mutant would not have been discovered at a higher temperature given its intrinsic inability to grow at higher temperatures.

In their study, Huang *et al.* determined that mutation of the Cdc50 lipid flippase β-subunit increased the sensitivity of this organism to caspofungin and fluconazole (Huang *et al.* 2016). By using a fluorescently labeled caspofungin molecule, they also observed that the *cdc50*Δ mutant strain displayed increased drug uptake compared to WT strains, suggesting that the altered membrane integrity and lipid content in the mutant strain might allow better penetration of the drug into the cell, and, therefore, higher efficacy. This observation implies that overcoming limitations of entry into or accumulation of the drug in the cryptococcal cell might enhance fungal cell killing. To further explore the importance of drug efflux, we assessed the effect of the efflux pump inhibitor clorgyline on caspofungin activity, and we found strong synergy between these two compounds. We specifically employed clorgyline as a pharmacological inhibitor of ABC transporters to address the potential functional redundancy of the large number of *C. neoformans* genes likely to encode efflux pumps (Loftus *et al.* 2005; Holmes *et al.* 2016).

In validation of our screening methods, we identified the calcineurin B subunit mutant as a hypersensitive strain. Calcineurin signaling is required for full virulence of *C. neoformans* and is involved in the response to elevated temperatures, as well as the response to caspofungin and other cellular stresses (Odom *et al.* 1997; Cruz *et al.* 2000; Del Poeta *et al.* 2000; Fox *et al.* 2001). Similarly, calcineurin signaling has been shown to be required for paradoxical growth in the presence of high concentrations of caspofungin in *Candida* species and *A. fumigatus* (Walker *et al.* 2008, 2012; Fortwendel *et al.* 2010; Lee *et al.* 2011; Juvvadi *et al.* 2015). In *C. neoformans*, calcineurin is known to regulate one transcription factor, Crz1, and many calcineurin-dependent cellular processes are likely mediated by Crz1 (Lev *et al.* 2012). Importantly, recent work identified several genes involved in cell wall biosynthesis and remodeling that are transcriptionally upregulated by Crz1 and Cna1 in response to thermal stress (Chow *et al.* 2017). However, this work also revealed that a significant number of genes are regulated differentially between the *cna1*Δ and the *crz1*Δ transcriptomes, suggesting that other targets of Cna1 activity could have effects on the transcriptional response to stress. Additional work demonstrated that calcineurin likely has multiple downstream targets in addition to Crz1, including proteins that localize to stress granules and P-bodies, and are involved in post-transcriptional responses to thermal stress (Park *et al.* 2016). Our work suggests that the calcineurin-regulated response to caspofungin likely involves both the activity of the Crz1 transcription factor as well as Crz1-independent calcineurin targets. Indeed, it has also been demonstrated in *C. albicans* that Crz1 is only partially responsible for the calcineurin-mediated echinocandin tolerance seen in that organism (Singh *et al.* 2009). Future work could probe Crz1-independent calcineurin targets for roles in caspofungin tolerance in this organism.

We also tested multiple processes regulated by the Pfa4 palmitoyltransferase to explore whether the drug-susceptible phenotype of the palmitoyltransferase mutant could be attributed to dysfunction of one or more of its downstream targets. Interestingly, we determined that the *C. neoformans* Ras1-mediated thermotolerance pathway is required for tolerance to caspofungin treatment in this organism. This signaling pathway responds to extracellular cues to control the activation of the Cdc42 GTPase, a protein that directs actin cytoskeleton polarization as well as directional growth and budding (Ballou *et al.* 2009). The septin proteins Cdc3 and Cdc12 are required for cytokinesis through their role in septum formation. Additionally, Cdc3 and Cdc12 are necessary for normal cell morphogenesis at elevated temperatures, and the localization of these proteins is mediated by Cdc42. In the absence of any of these pathway proteins, the cryptococcal cell is growth-impaired under many stressful conditions, such as mammalian physiological temperatures (Nichols *et al.* 2007; Ballou *et al.* 2009, 2013a). Although mutants of genes encoding two members of this pathway, *RAS1* and *CDC42*, were present in the collections tested in this study, their growth phenotypes are only apparent at elevated temperatures. As a result, these mutants would not be expected to display caspofungin susceptibility at the lower temperatures tested, emphasizing the importance of screening for drug sensitivity in multiple incubation conditions.

Since Ras and Ras-related proteins are required for pathogenesis in many fungal systems, there have been concerted efforts to identify small molecules that inhibit Ras protein function. One of the most promising directions includes targeting protein prenyltransferases, enzymes that add lipid moieties to the C-terminal regions of Ras-like GTPases, directing the localization of these proteins to cellular membranes, which are their sites of function (Vallim *et al.* 2004; Nichols *et al.* 2009; Fortwendel *et al.* 2012; Esher *et al.* 2016). Prenylation inhibitors are predicted to alter the localization and function of many cellular proteins in addition to Ras, potentially having broad antiproliferative effects in divergent cell types. Structural studies have identified fungal-specific features of farnesyltransferase enzymes, suggesting that antifungal specificity could be engineered into FTIs (Hast *et al.* 2011; Mabanglo *et al.* 2014). Antifungal susceptibility testing demonstrated *in vitro* synergy between the activities of caspofungin and the FTI manumycin A, the basis of which was identified in our screen. Importantly, the currently available FTIs tested have limited efficacy as antifungal compounds when used alone. However, as newer and more potent antifungal FTIs are identified, these agents might be used as adjunctive therapies to enhance the effect of caspofungin.

We also determined that chitin biosynthesis is upregulated during caspofungin treatment and that the Chs3 chitin synthase is required for caspofungin tolerance. As caspofungin inhibits the biosynthesis of the cell wall component β-1,3-glucan, upregulation of other cell wall biosynthesis or cell wall-modifying enzymes may compensate for altered glucan content. Indeed, in *A. fumigatus*, cell wall chitin deposition, as well as the expression of chitin synthase genes, increases during caspofungin treatment (Fortwendel *et al.* 2009, 2010). Additionally, *Candida* clinical isolates with naturally increased levels of chitin, as well as strains grown in chitin-inducing conditions, demonstrate increased survival during caspofungin treatment (Walker *et al.* 2008, 2012; Lee *et al.* 2011). A similar phenomenon appears to be occurring in *C. neoformans*: during caspofungin treatment, we measured increased levels of chitin both by CFW staining and by an *in vitro* biochemical quantification of chitin monomers. There is a similar increase in chitosan levels. As *Cryptococcus* species typically display higher levels of chitosan in the cell wall than other pathogenic fungal species (Banks *et al.* 2005), these inducible cell wall changes in chitin/chitosan content likely represent a conserved mechanism by which *C. neoformans* adapts to caspofungin treatment. Additionally, the increased baseline chitosan levels in the *C. neoformans* cell wall may also contribute to its innate tolerance to this drug.

In conclusion, several varied cellular processes were represented among the strains with enhanced caspofungin susceptibility. It is possible that the loss of some of these processes enhances the intracellular accumulation of the drug, preventing the low concentrations of caspofungin in *C. neoformans* cells suggested in prior studies. Alternatively, compromising these cellular pathways may render the *C. neoformans* cell susceptible to lower effective concentrations of this drug. As new fungal-specific inhibitors are developed for processes such as Ras protein localization and chito-oligomer synthesis, these agents may provide promising new directions for combination antifungal therapy.
